# Update on Calcitonin Screening for Medullary Thyroid Carcinoma and the Results of a Retrospective Analysis of 12,984 Patients with Thyroid Nodules

**DOI:** 10.3390/cancers15082333

**Published:** 2023-04-17

**Authors:** Martina Broecker-Preuss, Dietmar Simon, Mirka Fries, Elisabeth Kornely, Manuel Weber, Irfan Vardarli, Elena Gilman, Ken Herrmann, Rainer Görges

**Affiliations:** 1 Department of Medicine, Laboratory Medicine Section, Ruhr-University Bochum, University Hospital, Knappschaftskrankenhaus Bochum, 44892 Bochum, Germany; 2 Department of Endocrine Surgery, Bethesda Krankenhaus, Thyroid Center Rhine-Ruhr, 47053 Duisburg, Germany; 3 Clinic for Nuclear Medicine, University Hospital Essen, 45147 Essen, Germany; 4 Practice of Endocrinology, Thyroid Center Rhine-Ruhr, 47051 Duisburg, Germany; 5 Department of Medicine I, Klinikum Vest GmbH, Knappschaftskrankenhaus Recklinghausen, 45657 Recklinghausen, Germany; 6 5th Medical Department, Division of Endocrinology and Diabetes, Medical Faculty Mannheim, Heidelberg University, 68167 Mannheim, Germany; 7 Gilman Biometrics, Consultant for Data Processing and Statistics, 50858 Köln, Germany; 8 Practice of Nuclear Medicine, Thyroid Center Rhine-Ruhr, 47051 Duisburg, Germany

**Keywords:** medullary thyroid cancer, calcitonin, calcitonin screening, routine calcitonin measurement, nodular thyroid disease, calcium stimulation test

## Abstract

**Simple Summary:**

Medullary thyroid carcinoma (MTC) is a rare disease and accounts for about 5% of thyroid carcinomas. In contrast to other thyroid cancers, MTC can be detected by calcitonin (Ctn) measurement in blood samples. However, the interpretation of Ctn values is difficult since reference values for Ctn are sex-specific, assay-dependent, and can also be elevated in benign thyroid diseases and certain extrathyroidal conditions. Here, we report on the results of Ctn screening in 12,984 adult patients with thyroid nodules from our nuclear medicine practice. The Ctn values were elevated in 207 (1.6%) patients. Further clarification was possible in 124/207 cases, of which MTC could be ruled out in 108 cases. In 16/12,984 patients, MTC was confirmed. Thus, the extrapolated MTC prevalence is 0.14% in our patients. We thus recommend Ctn screening even in patients with very small thyroid nodules. High quality standards in pre-analytics, Ctn measurement, and the interpretation of data must be ensured.

**Abstract:**

Background: We provide an update on calcitonin (Ctn) screening for the early detection of medullary thyroid carcinoma (MTC) and present the results of a large single-center analysis evaluating sex-specific cut-off-levels and long-term courses. Methods: A total of 12,984 consecutive adult patients (20.1% male and 79.9% female) with thyroid nodules who had undergone routine Ctn measurement were retrospectively analyzed. Patients with confirmed suspicious Ctn values were referred for surgery. Results: Ctn measurements were elevated in 207 (1.6%) patients, with values below twice the sex-specific reference limit in 82% of these cases. Further clarification was possible in 124/207 cases, of which MTC could be ruled out in 108 cases. Histopathological assessment confirmed MTC in 16/12,984 patients. Conclusions: Our extrapolated MTC prevalence of 0.14% is significantly lower than that described in early international screening studies. The stimulation test can usually be dispensable when using a decision-making concept based on sex-specific basal Ctn cut-off values. Ctn screening is recommended even in patients with very small thyroid nodules. High quality standards in pre-analytics, laboratory measurements, and the interpretation of data must be ensured, as well as close interdisciplinary cooperation between medical disciplines.

## 1. Introduction

Calcitonin (Ctn) is a polypeptide hormone, the mature (monomeric) form of which consists of 32 amino acids with a disulfide bridge between positions 1 and 7, and it is mainly produced by the C-cells of the thyroid gland. Ctn has been established as a tumor marker for confirmed and treated medullary thyroid carcinoma (MTC) since the 1970s [1]. Since then, the measurement methods have continuously improved in terms of diagnostic sensitivity and specificity. In 2004, the Thyroid Section of the DGE (Deutsche Gesellschaft für Endokrinologie = German Society of Endocrinology) recommended routine Ctn measurement for the detection of MTC in patients with thyroid nodules [2]. The recommendation derived from international studies published between 1994 and 2002 in which the average MTC prevalence in the screening groups was determined to be 0.78% (evaluation of 8 studies with a total of 14,247 patients). The core argument for this recommendation was that MTCs can be detected biochemically more frequently in the early stages—usually before the occurrence of metastases—with a consecutive improvement in surgical results, in particular, an increased chance of curative intervention [3,4,5,6].

In the time since then, some general conditions have changed. In more recent Ctn screening studies, a significantly lower prevalence of MTC was described than in the initial publications (in the three published German studies only up to 0.2%), and the importance of sex-specific Ctn reference ranges has now been generally accepted for several years. In addition, since 2010/2011, pentagastrin is no longer approved or is no longer available as a stimulant for the differential diagnosis of equivocal (only moderate—up to about 50 pg/mL) basal Ctn elevations. Alternatively, the calcium stimulation test was suggested [7,8,9]. Yet, this test can also lead to false-positive results—for example in the case of papillary thyroid carcinomas (PTC) [10,11]—and its advantage of over-optimized basal Ctn threshold values has increasingly been questioned in recent years [12,13]. Because of these findings and different views on the health–economic analysis, there is no international consensus on Ctn screening to this day. Hence, screening is not generally recommended in the guidelines of various important professional societies, such as the current guidelines of the American Thyroid Association (ATA) [14,15].

MTC is certainly a rare disease. However, assuming that only 1–2 MTC cases per 1000 screened patients are being detected, its prevalence is higher than that of other established screening programs. For example, the epidemiological data for metabolic diseases, which in Germany must be recorded at the screening of newborns, reveal a prevalence of 1:3600 for congenital hypothyroidism (with a true-positive rate of 30% for an increased TSH value in the screening), 1:10,000 for phenylketonuria, 1:12,900 for adrenogenital syndrome, 1:68,000 for galactosemia, and only 1:160,000 for maple syrup urine disease. A study conducted in the USA [16] showed that Ctn screening in patients undergoing evaluation for thyroid nodules appears to be cost-effective, with cost-effectiveness comparable to TSH screening for congenital hypothyroidism, colonoscopy, and mammography screening.

We recently published a meta-analysis on the diagnostic accuracy of routine calcitonin measurement for the detection of medullary thyroid carcinoma in the management of patients with nodular thyroid disease [17]. This study was based on the published literature up to and including 2020 and yielded the following pooled estimates: sensitivity 0.99 (95% CI, 0.81–1.00), specificity 0.99 (95% CI, 0.97–0.99), positive likelihood ratio 72.4 (95% CI, 32.3–162.1), and negative likelihood ratio 0.01 (95% CI, 0.00–0.23). Since it has not been clarified whether data collected elsewhere can be easily transferred to each country or region (e.g., due to different epidemiological and environmental conditions) and the fact that the previous studies mostly did not consider sex-specific cut-off values, we carried out a retrospective mono-center study. Due to the very low MTC prevalence of <<1%, we included a large number of patients in the analysis to increase the statistical certainty of the data collected.

Our present study and paper pursued the following objectives:

1. To determine the spectrum of screening Ctn values and the current MTC prevalence in the Rhine–Ruhr area, the region with the highest population density in Germany;
2. To examine the impact of stimulation tests (especially the calcium stimulation test) vs. different, sex-specific basal Ctn cut-off values on Ctn screening;
3. To analyze the characteristics of the MTC cases detected by Ctn screening regarding Ctn values and sex distribution;
4. To understand and discuss the basic problems of Ctn screening for MTC.

In a second publication that will shortly be submitted for review, we will report on the surgical measures in MTC patients detected by Ctn screening and the outcomes. In particular, we will report ultrasonographic findings, TNM stages and germline RET proto-oncogene results, the significance of histologic findings of desmoplastic stromal reaction and analyze the correlation between tumor size and Ctn values.

## 2. Materials and Methods

### 2.1. Eligibility Criteria

For this retrospective study, we analyzed the database of the medical practice for nuclear medicine in Duisburg, Germany, focusing on all patients undergoing routine Ctn measurement due to thyroid nodules from November 2008 to November 2014. Patients who underwent surgery and recieved a histological diagnosis of medullary thyroid cancer and patients with unclear or borderline Ctn elevations were followed, if possible, until April 2022. The following inclusion criteria for Ctn screening were defined:

○ The presence of at least one thyroid nodule that is detectable by ultrasonography (down to about 2 mm size);
○ Age ≥ 18 years.

Exclusion criteria:

○ Advanced kidney disease *;
○ Known Ctn elevations **;
○ Personal or family history of thyroid carcinoma or MEN **;
○ Known mutations of the RET proto-oncogene **;
○ (* Because in the case of advanced renal insufficiency, non-specific increased Ctn values are often measured even with a Ctn assay, which detects the mature (monomeric) Ctn form with high selectivity; ** in order to avoid a preselection bias, which would simulate an unrepresentatively high MTC prevalence in the examined patients).

### 2.2. Ctn Measurement

Blood samples for routine Ctn measurement were collected in standard serum mono-vettes (an S-monovette with a clot activator, Sarstedt, Nümbrecht, Germany). The patients did not have to be fasted for blood collection. Information on factors that might influence Ctn levels (such as proton-pump inhibitor medication, Hashimoto’s thyroiditis, known neuro-endocrine tumors, comorbidities, and alcohol or nicotine use) was collected to explain any unspecific Ctn elevations. The serum monovettes were allowed to stand at room temperature for 20 min and were then centrifuged at 4 °C for 10 min with 2600× *g*. Aliquots of the serum in the supernatant were frozen in a separate tube at −20 °C until the Ctn measurement was performed.

Ctn measurements were routinely carried out using an immunoradiometric sandwich assay (IRMA) with two monoclonal antibodies against different epitopes of the Ctn molecule (SELco^®^ Calcitonin, MEDIPAN, Dahlewitz/Berlin, Germany). The reported LoD (limit of detection) is 1.6 pg/mL, the reported LoQ (limit of quantification, defined as the lowest analyte concentration that can be measured with an interassay coefficient of variation of 20%) is 3.0 pg/mL. The cut-off value for positivity was set by the manufacturer at 10 pg/mL for females and 15 pg/mL for males. The sex-specific reference ranges reflect the fact that the C-cell density in men is physiologically approx. 1 ½ times that of females with equivalent potential to release Ctn [18].

The plausibility of these recommended cut-off values was tested with samples from 40 men and 100 women with nodular thyroid disease who had not had previous thyroid surgery or radioiodine therapy but had undergone a thyroidectomy for various reasons after Ctn measurement (outside of this screening study), and in whom no MTC had been histologically detected (patients with only a partial or nearly total resection were not included). Reference intervals were calculated according to the CLSI C28-A3c guideline using the Abacus 3.0 software package (Abacus Validation Systems, Jena, Germany). Ctn values below the LoQ of 3.0 pg/mL were set to 1.5 pg/mL for the calculations. The Ctn reference ranges determined in this way for patients with nodular thyroid findings (90% confidence intervals) were <3.0 to 9.1 pg/mL for women and <3.0 to 14.1 pg/mL for men, i.e., very similar to the reference ranges specified by the manufacturer and with a highly significant sex difference (*p* < 0.0002).

For confirmation testing, serum aliquots were sent to the cooperating endocrinological practice of the Thyroid Center Rhine-Ruhr. Measurements were carried out there using a different immunoassay (IMMULITE^®^ 2000 calcitonin, Siemens Healthineers, Erlangen, Germany). This immunochemoluminometric assay (ICMA) had been recommended in the past as an adequate substitute for the (no longer available) automated assay by Nichols Institute Diagnostics, which had long been the reference test for highly specific measurements of the mature (monomeric) form of the Ctn molecule [19]. In the Immulite ICMA, the cut-off level for positivity is 8.5 pg/mL in males and 5.0 pg/mL in females, corresponding to the 95% confidence interval of the measured Ctn values of 120 male and 90 female healthy volunteers (measured Ctn range: up to 18.2 pg/mL for males and 11.5 pg/mL for females). Only for a short period of 4 weeks within the Ctn screening period (when the Ctn measurement in the nuclear medicine practice laboratory was not possible) were the serum samples sent directly to the endocrinological practice for analysis.

### 2.3. Stimulation Tests

For the stimulation tests, pentagastrin was the exclusive stimulant until 2009 and calcium gluconate has been the exclusive product since 2012. From 2010–2011, either product could be used. Measurements of stimulated Ctn were usually taken 0 (just before injecting the stimulant), 1, 2, 5, and 7 min after the intravenous administration of 0.5 µg pentagastrin/kg body weight or 2.5 mg calcium gluconate/kg body weight. (The late blood withdrawals were omitted in some tests.) The Ctn peak value was defined as the maximum Ctn value measured during the test. The stimulation tests were carried out in the cooperating endocrinological practice (Ctn measurements with the ICMA).

Ctn peak values of 100 pg/mL (male patients) and 50 pg/mL (female patients) were defined as cut-off values for the pentagastrin stimulation test; 131 pg/mL (males) and 90 pg/mL (females) were defined as cut-off values for the calcium stimulation test [20].

### 2.4. Procedures for Clarifying Increased Ctn Values

**Figure 1:**
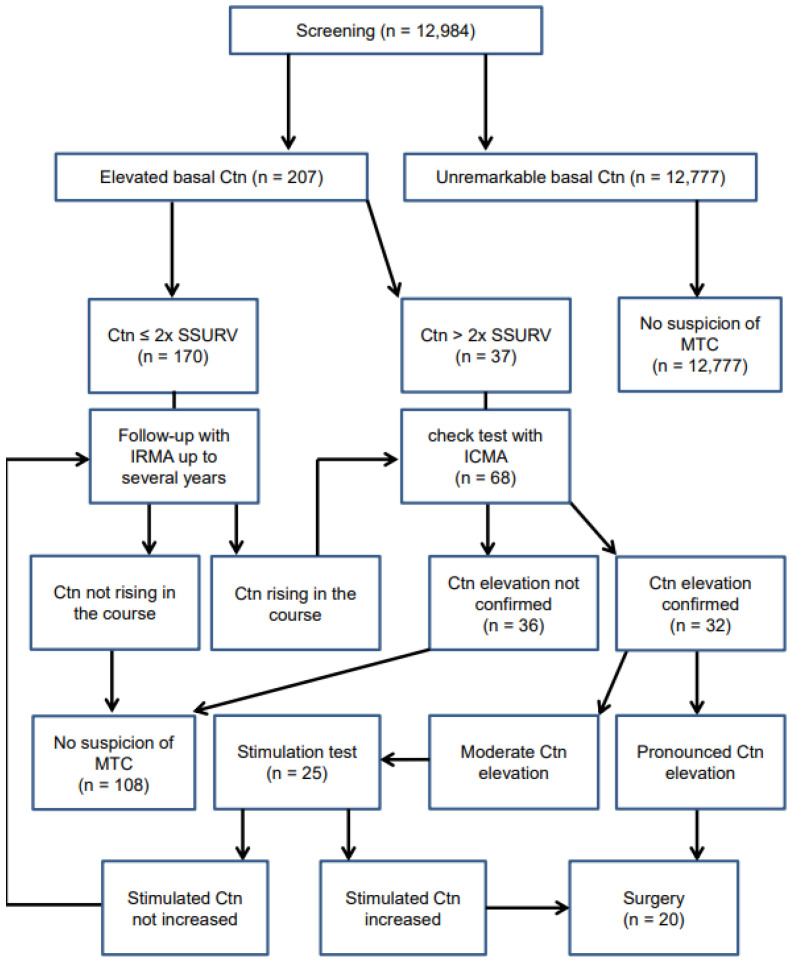
Flow chart with the common diagnostic algorithm in the context of the routine Ctn measurement of our study (deviations from this scheme were possible in individual cases). SSURV = sex-specific upper reference value.

If the Ctn values were only slightly increased, i.e., within a range of twice the sex-specific upper reference values (SSURV), which is up to 20 pg/mL in women and up to 30 pg/mL in men, the next step was usually a follow-up test with the same IRMA (SELco Calcitonin, Medipan). In case of more pronounced Ctn elevations or increasing Ctn values in the further course, a serum sample was usually sent to the collaborating endocrinological practice for a confirmation test with the ICMA. Carcinoembryonic antigen (CEA) was measured in parallel. Depending on the degree of Ctn elevation, patients with confirmed elevated Ctn values either had a follow-up basal Ctn measurement 3–12 months later and/or a stimulation test, or they were immediately referred for surgery (see the flow chart, Figure 1).

Patients with nodules ≥1 cm on ultrasonography received thyroid scintigraphy, according to the practice guideline of the European Association of Nuclear Medicine [21] regardless of the current TSH value since in iodine deficiency regions such as Germany, the TSH value has only a low predictive value for the functional status of a thyroid nodule [22,23,24]. In selected cases of scintigraphically non-autonomous nodules, a fine-needle biopsy was performed. If there is a strong suspicion of MTC, we usually performed no FNB, since FNB can induce fibroblastic proliferation, which can mimic tumor-induced a desmoplastic stromal reaction (DSR) and thus removes the basis for corresponding surgical concepts based on intraoperative frozen section diagnostics, since the extent of surgical intervention in MTC is increasingly being made dependent on evidence of a DSR. Moreover, if the Ctn elevation suggested the presence of a tumor, this result could not be invalidated by those aforementioned procedures: the suspected diagnosis of a C-cell neoplasia (CCN) could only be refuted with inconspicuous histology after total thyroidectomy or with inconspicuous results of Ctn controls. Patients who were screened with (usually only moderately) elevated Ctn values but who had not undergone surgery were followed at most until April 2022.

An elevated basal Ctn value was rated as unspecific or false-positive if at least one of the following criteria was met:

○ Ctn drops during follow-up or no increase within an observation period of several years;
○ The confirmation test with the monomer-specific ICMA showed an inconspicuous Ctn value;
○ Inconspicuous Ctn behavior in a stimulation test;
○ Total thyroidectomy without histopathological evidence of MTC (if only a partial thyroid resection was performed without histopathological demonstration of a CCN, this was not considered as an MTC exclusion).

### 2.5. Postoperative Assessment of Confirmed MTC Cases

Postoperative assessment entailed histopathological evaluation and tumor staging pursuant to AJCC/UICC guidelines, RET proto-oncogene germline analysis, and clinical follow-up. The latter included a physical examination, high-resolution ultrasonography of the neck, and laboratory diagnostics, in particular Ctn and CEA measurements, with a follow-up frequency of once or twice a year. The follow-up of the patients lasted at most until April 2022.

### 2.6. Statistics

All statistical analyses were performed with IBM SPSS version 26 (IBM, Armonk, New York, NY, USA). Categorial variables were shown as absolute or relative frequencies and the Chi^2^-Test (Fisher-Test for 2 × 2 tables) was used to test the significance. Numeric variables were expressed as the mean and standard deviation; in the case of skewed distribution, the median and range were used. A t-test was used to compare two normally distributed numeric variables; non-normally distributed variables were compared using the Mann–Whitney U test. The correlations between non-normally distributed variables were computed using Spearman’s method. All results were regarded as significant at a *p*-level of <0.05.

## 3. Results

### 3.1. Spectrum of Screening Ctn Values and MTC Prevalence

**Figure 2:**
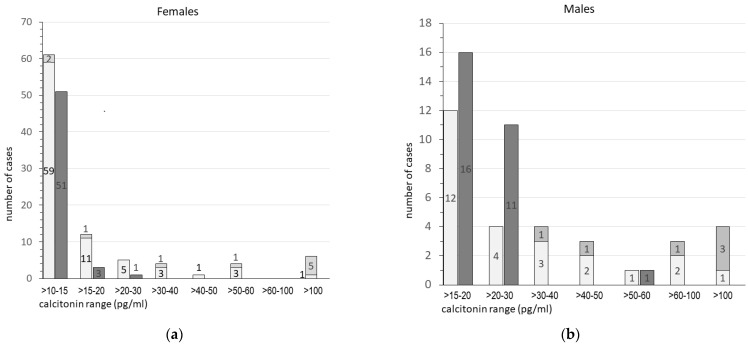
(**a**,**b**). Histograms of the distribution of the Ctn values above the sex-specific reference ranges (female patients = left side; male patients = right side). White columns: patients in whom MTC could be excluded; light gray columns: patients with confirmed MTC; dark gray columns: patients in whom no further clarification of the Ctn elevation was possible. Considering the distribution of the Ctn values, the statistical extrapolation showed that in 2 additional female patients of the 83 patients with increased Ctn values for whom no further clarification was possible (dark gray columns), MTC could be expected.

During the screening period from 11/2008 to 11/2014, routine Ctn measurement was carried out in a total of *n* = 12,984 patients (m:f = 1:3.98) with nodular thyroid findings in ultrasonography. In 207/12,984 (1.6%) of the patients screened (*n* = 148 females and *n* = 59 males), these basal Ctn measurements yielded values above the normal sex-specific ranges of the IRMA (up to 10 pg/mL in women and up to 15 pg/mL in men). The histograms in Figure 2 show the distribution of the elevated Ctn values from the entire patient population that was screened.

In 83 of the patients with elevated Ctn values during the screening period, we were not able to perform further controls, because they no longer visited our institution (despite attempts to contact them), and no information on their further progression could be obtained from the referring general practitioners. However, the Ctn values of these patients were relatively low (range: 15.1–57.7 pg/mL in the male patients and 10.1–25.9 pg/mL in the female patients). The values were always below the sex-specific cut-off limits to recommend thyroidectomy and—except for one male and one female patient who had the highest values—were only within twice the SSURV, i.e., even below the lower threshold proposed by the DGE [12].

Further clarification was possible in 124 of the patients with elevated Ctn values. In 108/124 patients with Ctn values above the normal sex-specific ranges, MTC was ruled out, primarily because of unremarkable results of the confirmation test with the Immulite ICMA and/or due to non-increasing or even decreasing Ctn values with the IRMA, mostly over a follow-up period of several years. In nine patients, a complete thyroidectomy was performed for various reasons without histological evidence of MTC.

In 20 patients with increased basal Ctn values, further diagnostic procedures underscored the suspicion of a C-cell neoplasia of the thyroid gland and, for this reason, total thyroidectomy with stage-appropriate lymph node dissection was performed. MTC was found in 16 cases, while only C-cell hyperplasia (CCH) was detected in three cases and PTC only, but no MTC in one case. These cases and the indications for surgery are summarized in Table 1. Based on these numbers, the MTC frequency in our screening cohort was calculated as 0.12% (16/12,984). For the 83 patients with increased Ctn values for whom no further clarification was possible, we calculated the expected MTC prevalence by means of statistical extrapolation. The calculation was based on the distribution probability of the Ctn values and resulted in two additional female patients with probable MTC. Based on these calculations, the extrapolated overall MTC prevalence was 0.14% (18/12,984).

Regarding the sex distribution of the 124 patients with elevated Ctn values, for which further clarification was possible, *n* = 93 patients were female and *n* = 31 were male. MTC could be ruled out in 83/93 of the female patients and 25/31 of the male patients. Using the above data and based on the sex-specific reference ranges of the IRMA, false-positive results of Ctn screening could be extrapolated in 132/148 (89%) of the female patients and 48/59 (81%) of the male patients with elevated Ctn, i.e., in 1.3% of the female patients and in 1.8% of the male patients screened in total.

### 3.2. Stimulation Tests vs. Sex-Specific Basal Ctn Cut-Off Values

During the Ctn screening study period, stimulation tests were carried out on a total of 25 patients: in 5 patients with pentagastrin and in the remaining 20 patients with calcium gluconate. In Table 1, data from the 20 patients in whom thyroidectomy was performed on the basis of the examination findings are listed.

All 16 patients with histologically confirmed MTC showed an unequivocal pathological stimulability. In three male patients with significantly increased Ctn peak values (703 or >2000 pg/mL after calcium gluconate and 450 pg/mL after pentagastrin), the histological examination did not reveal an MTC, but a PTC in the first case and a CCH in the latter two cases.

**Figure 3:**
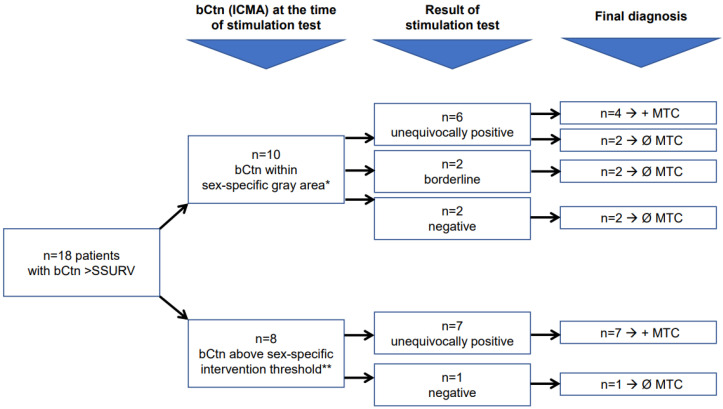
Comparison between basal Ctn values (bCtn, measured with the ICMA at the time of the stimulation test) and the results of the calcium gluconate stimulation tests. SSRUV = sex-specific upper reference values; * sex-specific gray area: SSRUV—30 pg/mL in females, SSRUV—60 pg/mL in males; ** sex-specific intervention threshold: 30 pg/mL in females and 60 pg/mL in males.

To retrospectively compare the diagnostic contribution of the calcium gluconate stimulation test versus the use of basal sex-specific Ctn cut-off values, 18 patients (10 f, 8 m) were identified in whom a calcium gluconate stimulation test had been performed and either a histological diagnosis after total thyroidectomy or long-term basal Ctn (bCtn) follow-up measurements were available. The results are visualized in Figure 3 and are explained in detail in the following text.

In 10 of the above 18 patients (5 f, 5 m), the ICMA measured bCtn values at the time of the stimulation test that were above the SSURV but did not exceed 30 pg/mL in females and 60 pg/mL in males:

- In 6/10 patients, the stimulation with calcium gluconate was strongly positive and led to total thyroidectomy, whereby histopathological examination revealed MTC only in four patients (peak stimulated Ctn (sCtn) values between 612 and >2000 pg/mL). The follicular variant of a PTC (m, bCtn 31.7 pg/mL at the time of the stimulation test and peak sCtn 703 pg/mL) or a CCH (m, bCtn 50 pg/mL and peak sCtn > 2000 pg/mL) was only seen in the remaining two patients.
- In 2/10 patients, the test produced no suspect Ctn stimulation (peak sCtn values were 9.9 and 64.0 pg/mL, respectively; MTC could be excluded in one case by non-increasing bCtn values during follow-up; in the other case MTC was histologically excluded).
- In another 2/10 patients, the test only produced a Ctn increase in the borderline pathological range. In two female patients, Ctn was increased from basal 10.9 to a maximum of 187 pg/mL (histologically no MTC but only CCH was detected, but the initial screening value was 51.6 pg/mL in the IRMA and 65.6 pg/mL in the ICMA) or from 10.9 pg/mL to a maximum of 180 pg/mL (in the long term, however, no increase in the bCtn values was observed).

In the remaining 8 patients of the above 18 patients with the calcium gluconate test (5 f, 3 m), the ICMA-measured bCtn values above the intervention thresholds recommended by the DGE (>30 pg/mL in women and >60 pg/mL in men). Only in one of these patients did the stimulation test not provide a pathological result (f; bCtn 42 pg/mL and peak sCtn 77.5 pg/mL with the ICMA); therefore, surgery was omitted in this patient. Follow-up tests within an 8-year observation period showed no increase in bCtn values. All of the other seven patients had significantly increased sCtn values (peak values 459 to >2000 pg/mL), and MTC was histopathologically confirmed after total thyroidectomy in each case. In five of these seven patients, bCtn values >100 pg/mL were measured.

Thus, based on the analysis of the subgroup of patients whose basal Ctn values were above the sex-specific intervention thresholds recommended by the DGE, only one had a “false-positive” bCtn value but a true-negative sCtn value (in another patient whose Ctn value was above the intervention threshold in the initial screening, the bCtn value at the time of the stimulation test was only slightly above the SSURV, but the sCtn value was also slightly increased). In the subgroup of patients whose basal Ctn values were above the SSURV but below the sex-specific intervention thresholds recommended by the DGE (“gray area”) at the time of the stimulation test, the calcium gluconate test yielded false-positive results in 2/6 patients with significantly increased sCTn values.

It is noteworthy that two of the histologically confirmed MTC patients with true-positive stimulation test reactions had already been identified several years earlier in the initial Ctn screening. Then, their bCtn values had been only slightly above the SSURV threshold: patient MTC-16 (f, initial Ctn screening value 10.1 pg/mL) only presented again 3.75 years later and patient MTC-09 (f, initial Ctn screening value 14.6 pg/mL) came 5.1 years later for the stimulation test. At the time of re-presentation, the bCtn values of these patients were 25.1 pg/mL (peak sCtn > 2000 pg/mL) and 71.6 pg/mL (peak sCtn > 2000 pg/mL). While in both cases, the MTC would have been detected earlier by the stimulation test; due to the increase in the bCtn values over time, it was still recognized early enough.

### 3.3. Characteristics of the Confirmed MTC Cases

In the 16 MTC patients identified during the screening study period, the male:female ratio was 1:1.67; thus, the proportion of males was higher (*p* = 0.110, n.s.) than in the whole population that was screened (*n* = 12,984; m:f = 1:3.98). The median age of the male MTC patients at the time of surgery was 72 years (range: 44–77 years) and of the female patients, it was 56 years (range: 29–78 years; *p* = 0.263, n.s.).

The basal Ctn values measured in the screening ranged from 30.5 to 1132 pg/mL in male MTC patients (median 158.7 pg/mL) and from 10.1 to 1276 pg/mL in female MTC patients (median 90.5 pg/mL). The difference between the men and women (in this relatively small sample) did not reach a level of statistical significance (*p* = 0.713). The carcinoembryonic antigen (CEA) was additionally determined in 14 of the MTC patients at the time of the first screening: values above the reference range (up to 3.8 ng/mL) were found in 9/14 (64%) with a range from 4.1 to 42.2 ng/mL (Supplementary Table S1).

In 5/16 MTC patients (MTC-03, MTC-04, MTC-09, MTC-13, and MTC-16), the basal Ctn values measured at the initial screening were below the values that the DGE had proposed as cut-off values for a recommendation for thyroidectomy. Three of these patients underwent surgery shortly after Ctn screening due to the highly elevated peak Ctn values in the calcium stimulation test. The other two (MTC-09 and MTC-16, female patients) only presented in our institution again after more than five and three years, respectively (as already described in the last paragraph of Section 3.2). At that time, their basal Ctn values were 71.6 and 25.1 pg/mL, respectively, and they also had highly increased peak values in the calcium stimulation test, so surgery was performed.

## 4. Discussion

In 2004, the DGE recommended Ctn screening for the detection of MTC in nodular goiter [2], but without further comments regarding the size and features of the existing nodules or the planned therapeutic measures. Studies on the cost–benefit ratio or health–economic analyses on Ctn screening were not available at that time. Currently, such studies are still incomplete or only available for a few countries/health systems. The German Society for General and Visceral Surgery (DGAV) also adopted the recommendation for Ctn measurement in nodular thyroid diseases in its current S2k guideline for the surgical treatment of benign thyroid diseases [25], at least before a planned thyroid surgery. In contrast to settings in which Ctn measurement is only carried out in the event of a planned surgery, the implementation of the recommendation made by the DGE to perform routine Ctn measurement in all patients with thyroid nodules creates a data situation that enables us to analyze the true impact on Ctn measurement more validly.

The recommendations of other international specialist associations vary on this point. According to the guidelines of the European Thyroid Association (ETA) jointly with the American Association of Clinical Endocrinologists (AACE) and the Associazione Medici Endocrinologi (AME), basal Ctn measurement may be considered in nodular thyroid disease and is especially recommended if fine-needle aspiration (FNA) biopsy results are suggestive of MTC or in patients undergoing thyroid surgery. It is mandatory in patients with a family history or clinical suspicion of MTC or multiple endocrine neoplasia 2 (MEN 2) [26]. In the current ATA guideline, there is no positive consensus recommendation for Ctn screening [14,15].

To the best of our knowledge, we are publishing the first study on Ctn screening carried out in Germany, which was based on sex-dependent reference ranges and cut-off values. It is one of the largest single-center studies on this topic worldwide that is evaluating long-term courses. Three previous German studies on routine Ctn measurement used sex-independent cut-off values, as have most international studies [17]: Rink et al. [27] and Herrmann et al. [28] used a Ctn threshold of 10 pg/mL and Schneider et al. [29] used a Ctn threshold of 13 pg/mL. Values of 1½ to 2 times higher male than female reference ranges and threshold values are now generally accepted in both adults and children [30,31,32,33] and reflect the fact that the density of thyroid C cells is about twice as high in male subjects compared to female subjects [18,30].

Based on the sex-specific reference ranges of the IRMA (up to 10 pg/mL for women and up to 15 pg/mL for men), we calculated the rate of false-positive Ctn elevations with 1.3% for the female patients and 1.8% for the male patients screened in total. Previous studies [27,28,34,35,36,37,38,39] defined the maximum reference range for both women and men at up to 10 pg/mL. Since the lowest Ctn screening value in our study was 30.5 pg/mL for men and 10.1 pg/mL for women with confirmed MTC, raising the male Ctn reference range to 15 pg/mL reduced false-positive Ctn elevations but did not result in missing true MTC cases.

Thus, the justification of a sex-dependent approach in Ctn screening is supported by our current study results. Furthermore, in our control group with nodular goiter without histological evidence of MTC after thyroidectomy, we found that men had significantly higher Ctn values than women. In this control group, 3% of women had Ctn levels above 10 pg/mL and 3% of men had Ctn levels above 15 pg/mL (in the sense of false-positive Ctn elevations); lowering the upper male reference limit to 10 pg/mL (identical to the female reference range) would increase the false-positive rate in this collective to 16%. The bCtn values in men (median and range) were also higher in all histologically verified MTC patients than in women, although the level of statistical significance was not reached, possibly due to the relatively low number of cases.

The sex ratio of the total of 12,984 screened patients in our study population (m:f = 1:3.98) is similar to most other published studies (see Table S2, Supplementary Materials). With regard to sex distribution, we found differences between the total number of patients screened and the 207 patients whose bCtn values exceeded the sex-specific reference range (m:f = 1:2.51) and between the latter 207 patients and the 16 ultimately confirmed MTC cases (m:f = 1:1.67). However, these differences were not statistically significant.

### 4.1. Spectrum of Screening Ctn Values and MTC Prevalence

Table S2, Supplementary Materials, compares the basic parameters and the MTC prevalence identified in various international Ctn screening studies with our results (only studies with a number of >1000 cases were taken into account). The frequency of MTC cases detected by means of routine Ctn measurement in patients with nodular thyroid glands of at least 0.12% (max. 0.14%, including the statistically expected 2 MTC in the group of unclarified cases) corresponds well with previous studies carried out in Germany: Rink et al. [27] reported 28/21,928 (0.13%), Herrmann et al. [28] reported 2/1007 (0.2%), and Schneider et al. [29] reported 10/11,270 (0.09%).

In a systematic review of autopsy series from 1970 to 2006, an average prevalence of 0.14% for occult MTC was found in 7897 autopsies from 24 published series from 21 different countries [40]; in nearly all cases, the tumor size was smaller than a centimeter without evidence of lymph node spread, extrathyroidal extension, or distant metastases. This prevalence is comparable to the MTC prevalence determined in German Ctn screening studies.

All of these data indicate that the actual prevalence of MTC in our country is significantly lower than the prevalence of 0.6% and more, which was reported in early international studies [34,41,42]. This fact should be included in considerations regarding future recommendations for the implementation of Ctn screening—in which health–economic aspects must always be considered.

### 4.2. Stimulation Tests vs. Sex-Specific Basal Ctn Cut-Off Values

In the past, calcitonin stimulation tests with pentagastrin and/or calcium gluconate were recommended as the standard for further clarification of suspected MTC if basal Ctn measurements had yielded values in the only slightly increased (“gray”) area [2,43,44]. Using these tests should help to differentiate patients with non-tumor-related Ctn elevations (e.g., due to CCH) from MTC cases which would improve the planning of optimal further care. In the German Association of Endocrine Surgeons practice guidelines for the surgical management of malignant thyroid tumors [43], total thyroidectomy is generally warranted if stimulated calcitonin serum levels are higher than 100 pg/mL in adults since the risk of MTC is considered to be substantial if this cut-off value is exceeded. Since 2010/2011, pentagastrin has become increasingly difficult to obtain/has not been available, and the Thyroid Section of the DGE initially suggested a return to the calcium gluconate test [8] which had already been used since the 1970s.

In recent years, there has been an increasing number of publications that focus on establishing optimal sex-specific bCtn cut-off values to separate patients without C-cell pathology and CCH cases from MTC patients as best as possible. The results from several studies with various underlying cut-off values—ranging from >18.7 to ≥35 pg/mL in females and >34 to >68 pg/mL in males—are summarized in Table 2.

In 2018, the Thyroid Section of the DGE recommended that further procedures in Ctn screening should be made dependent only on the level and/or the development of sex-specific basal Ctn values and that stimulation tests should be largely omitted [12]. The arguments for this are that the sensitivity and the specificity of the Ctn assays that are available nowadays have highly increased, the effort and potential for side effects of the calcium gluconate test are high, and the cut-off values of the calcium gluconate test for diagnosing MTC are not well evaluated, especially concerning the risk of false-positive results. Furthermore and, above all, reliable study data are being published meanwhile that confirm the clinical relevance of various levels and the dynamics of basal Ctn values.

Thus, some unspecific causes of bCtn elevations can be better ruled out nowadays. It is true that there is still an overlap area in which it is not possible to reliably distinguish between CCH and MTC based on single bCtn values. However, this shortcoming can be overcome through follow-ups every 3–6 months, during which a further increase in bCtn values indicates an MTC and should be clarified surgically. The Thyroid Section of the DGE proposes a gray area of 20–30 pg/mL for females and 30–60 pg/mL for males [12]. If the bCtn values exceed the proposed gray areas, surgery is generally recommended because higher values not only increase the probability of MTC, but also the risk that lymphogenic metastatic spread has already occurred. Niederle et al. [13] found cut-off levels of >85 pg/mL for females and >100 pg/mL for males for the prediction of lateral neck lymph node metastasis).

However, some authors still recommend the calcium gluconate test as a helpful tool for diagnosing MTC. A recent Italian study [45] postulated that a combination of optimized basal and calcium-stimulated Ctn thresholds is required to achieve maximum diagnostic accuracy. Outside of routine cases, there are special constellations where the importance of stimulation tests is less controversial: these include increased basal Ctn values with suspected MTC in patients with severe kidney disease, especially those requiring dialysis [46] and the differentiation of extrathyroidal Ctn-releasing neuroendocrine tumors [47].

The analysis of our patient subgroup that had a calcium gluconate stimulation test and either a histological diagnosis after total thyroidectomy, or for whom long-term bCtn follow-up results were available, showed no clear superiority of the stimulation test compared with the DGE recommendations based on basal Ctn values. Although the MTC diagnosis would have been recognized earlier in some patients with a stimulation test, the preoperative long-term courses of patients MTC-09 and MTC-16 show that this diagnosis would also have been reached based on the increased basal Ctn values. Admittedly, the diagnosis would have been established later but without disadvantages for the patients. On the other hand, the calcium gluconate stimulation test gave false-positive results in 2/6 cases with markedly increased sCtn values and thus led to unnecessary operations. Conversely, in the 11 analyzed patients from the subgroup with bCtn values above the sex-specific intervention thresholds recommended by the DGE, there was also 1 patient who was falsely positive but truly negative in the stimulation test. In one patient without MTC, both the bCTn measurement and the stimulation test yielded equivocal (borderline) results.

**Table 2:** Overview of sensitivity, specificity, positive predictive values (PPV), and negative predictive values (NPV) of sex-specific bCTn cut-off values, which were determined in various studies to differentiate between patients without C-cell pathology and CCH cases on the one hand and actual MTC- cases on the other hand.

	**Females**				
**Source**	**bCtn Cut-Off**	**Sensitivity**	**Specificity**	**PPV**	**NPV**
Colombo et al., 2012 [7]	>18.7 pg/mL	100%	100%	100%	100%
Mian et al., 2014 [48]	>26 pg/mL	81.8%	97.9%	94.7%	92%
Allelein et al., 2018 [49]	≥35 pg/mL	87.3%	87.5%	98%	50%
Niederle et al., 2020 [50]	>23 pg/mL	81%	100%	100%	83%
Fugazzola et al., 2021 [45]	>30 pg/mL	75.9%	93.7%	85%	86.5%
	**Males**				
**Source**	**bCtn Cut-Off**	**Sensitivity**	**Specificity**	**PPV**	**NPV**
Colombo et al., 2012 [7]	>68 pg/mL	100%	100%	100%	100%
Mian et al., 2014 [48]	>68 pg/mL	83.3%	100%	100%	92.9%
Allelein et al., 2018 [49]	≥46 pg/mL	93.6%	95%	97%	90%
Niederle et al., 2020 [50]	>43 pg/mL	53%	100%	100%	83%
Fugazzola et al., 2021 [45]	>34 pg/mL	88.9%	95%	88.9%	92.7%

### 4.3. Basic Problems of Ctn Screening for MTC

#### 4.3.1. The Problem of False-Positive Cases

Numerous factors have been described to cause (mainly moderate) Ctn elevations even though no MTC is present [32,47]. Heterophilic antibodies can cause a direct linking between capture and detector antibodies in the assay and thus a false-positive measurement signal without “sandwich formation” with the specific antigen [51]. In addition, various circumstances (e.g., otherwise malignant processes involving the thyroid gland, autoimmune thyroid disorders, previous thyroid surgery, hyperparathyroidism, and hypercalcemia of various origins) are known to result in reactive, non-neoplastic CCH. The latter can usually only be differentiated from neoplastic CCH, i.e., a precancerous condition, by molecular genetic diagnostics (mutation analysis of the RET proto-oncogene). Significantly increased Ctn values sometimes occur with extrathyroidal neuroendocrine tumors, also with other extrathyroidal diseases, for example of the kidneys (especially in advanced renal insufficiency or those on dialysis) as well as with severe lung and liver diseases.

Only a few of those factors discussed in the literature that may cause an “unspecific” Ctn increase could be determined in a small proportion of our patients with false-positive Ctn values. The most commonly identified factors in patients with nonspecific Ctn elevations were concomitant autoimmune thyroid disease or the use of proton pump inhibitors (PPIs), although the presence of these factors was no more common in patients with nonspecific Ctn elevations than in patients with normal Ctn values. This finding justifies our approach of not categorically excluding patients with Hashimoto’s thyroiditis and PPI intake from the Ctn screening study, in contrast to some other studies [29,38]. Most recent publications that used modern immunometric assays negated a relevant effect of Hashimoto’s thyroiditis as a cause of increased basal Ctn values as well as PPI therapy [32,37,52]. For us, this means that the reason for “false-positive” Ctn values could not be indisputably established for most of our affected patients.

#### 4.3.2. The Problem of True- and False-Negative Cases

As with almost all previous studies, we did not conduct systematic examinations of the specific rate of false- and true-negative cases in patients with basal Ctn values within the sex-specific reference ranges. This rate is generally considered to be very low. Ideally, such an examination would be a histological exclusion of MTC in a representative number of patients with normal Ctn values as a result of processing the entire resected tissue from a total thyroidectomy by means of thin layer serial sectioning (see the “Gold Standard” section below). However, we have an overview of the histological findings from more than 100 patients whose routine Ctn values were within reference ranges during our screening study. These patients had had a thyroidectomy for other reasons, and in none of the cases was a previously unsuspected MTC found.

In our recently published meta-analysis on Ctn screening [17], we only found a few studies in which the histological findings of some patients who had undergone thyroid surgery despite normal Ctn values were reported. For example, Schneider et al. [29] reported two patients with incidentally diagnosed MTC who had false-negative basal Ctn values. In these few MTC cases in patients who had no pathological screening, the Ctn values were assessed as false-negative in our meta-analysis. Because of the extreme rarity of such cases, it seemed legitimate for the meta-analysis to set the false-negative rate to nearly zero in studies in which false-negative cases were not reported.

False-negative screening results that lead to suboptimal pre-analytics and ultimately to artificially low Ctn values should of course always be ruled out in institutions performing Ctn measurements. Hook effects that can lead to falsely negative values in the presence of extremely high Ctn concentrations [53,54], should hardly ever occur with the two-step immunometric assays commonly used today. This is because the intermediary washing steps remove the Ctn molecules not bound to the capture antibodies before detector antibodies are added. Apart from MTC that do not release Ctn and are immunohistochemically Ctn-negative [55,56], even immunohistochemically Ctn-positive MTC can be associated with Ctn levels that are in the reference range or below the cut-off values. This might be the case if the tumor mass is very small at the time of screening or if the tumor is able to produce but not secrete the calcitonin protein [56,57]. In a bicentric study with 839 patients with sporadic MTC, a prevalence of non-secretory MTC of only 0.83% of patients with MTC was found [58].

We repeated the Ctn measurement after about 5–6 years in numerous patients who presented for follow-up checks of their nodular goiter. All suspicious Ctn elevations and histological MTC diagnoses were reliably recorded at this point. However, in none of the MTC cases that were detected 5 years after the initial screening period could the previous Ctn values within the reference range be measured. This finding corresponds well with the results of a recently published study [59], in which the courses of 170 patients with nodular thyroid and Ctn values ≤10 pg/mL in initial screening were followed. None of these patients had a suspected Ctn increase or histopathologic evidence of MTC over a median follow-up of 53.0 months (range 23.9–102.5). With larger screening populations, “false-negative cases” will of course occur in isolated patients. In our study, the initial Ctn screening values in patients MTC-09 and MTC-16 were only slightly above the maximum reference range value and would probably have been completely unremarkable if the examination had been carried out 1–2 years earlier.

#### 4.3.3. The Problem of Assay Dependency of Measured Ctn Values

Nowadays, Ctn measurement should be carried out as standard using immunometric two-site sandwich assays, which work with both a monoclonal capture and detector antibody that are directed against different defined epitopes of the Ctn molecule. This guarantees that the monomeric (mature) form of the Ctn molecule is measured with high selectivity and that the rate of increased Ctn values of non-specific genesis should be minimized. These values could be the result of isoforms (e.g., polymeric forms and precursors) of Ctn. The LoQ of the assay used should be able to reliably depict the Ctn concentrations of the middle sex-specific reference ranges of the normal population.

Although the IRMA routinely used in our study meets these criteria, a previous study [60] has shown that it is somewhat more susceptible to unspecific influences such as procalcitonin levels, PPI intake, and, above all, chronic kidney disease (in contrast, not significantly in Hashimoto’s thyroiditis) as compared to the Immulite ICMA. For this reason, if the Ctn values we measured were elevated at more than 2 × SSURV or had not yet exceeded this threshold but showed an increase over time, we performed a confirmation test with the Immulite ICMA. This was necessary for 68 patients. These control measurements with the Immulite ICMA did not confirm the Ctn elevations in 36/68 cases. When the subsequent basal Ctn values of these patients were monitored with the IRMA over several years after the initial screening, no further increase was seen.

We may conclude that the percentage of Ctn values that moderately exceeded the maximum value of the reference range would only be half as high (due to the lower rate of unspecific Ctn elevations) if the Immulite ICMA had been used primarily in the screening instead of the IRMA. Nevertheless, the total proportion of IRMA-Ctn values above the reference ranges was 1.6% in our study. This result matched previous large screening studies listed in Table S2, Supplementary Materials, in which this percentage ranged from 0.3% to 5.9% (median 3.8%). However, most other studies did not use sex-specific reference ranges, and an enhancement of the upper reference values for male patients would have reduced the proportion of values classified as increased in those studies.

It should be emphasized that the Ctn values which were found to be nonspecifically elevated only with the routine IRMA (and thus false-positive in terms of an MTC diagnosis) were almost always below the proposed intervention threshold values of 30 pg/mL for women and 60 pg/mL for men. Therefore, they would not pose a serious threat with regards to the thresholds, since further follow-up controls would have to be carried out in these cases.

#### 4.3.4. The Problem of Histopathological Examination as the “Gold Standard”

In most studies, the histological evidence of MTC is considered as the gold standard for assessing the accuracy of a Ctn measurement. If a thyroidectomy is performed under suspicion of MTC due to increased Ctn values, this must be communicated to the pathologist. In such a case, the pathological processing of the resected material must be much more extensive than after other thyroid operations (by means of thin layer serial sectioning), since the primary tumors are often only a few millimeters in size and cannot be localized preoperatively. It is not uncommon that the resected thyroid gland must be processed completely. The immunohistochemical detection of Ctn is also mandatory.

To underline this statement, three of the ultimately confirmed MTC cases in our screening study were initially not diagnosed at the pathological institution that had been primarily involved (patients MTC-01, MTC-04, and MTC-16). In these cases, only the repeated examination of the resected material in the German pathology reference laboratory for thyroid tumors led to the confirmation of the suspected MTC diagnosis. In three other male patients with only moderately elevated bCtn screening values (17.8 and rising over time/35.5/47.2 pg/mL) but significantly increased sCtn values (peak 703 pg/mL/>2000 pg/mL, each after calcium gluconate, and 450 pg/mL after pentagastrin), no MTC could be detected even in the reference pathological analysis of the sample material submitted (the histological examination revealed CCH in the first two cases and PTC in the third case). However, there is no reliable information on whether the entire resected thyroid of these three patients was available for the second examination.

From these findings, we can derive the requirement that an in-house pathology department that does not necessarily have to be highly specialized in thyroid malignancies should aim for a reference pathological assessment in the event of discrepancies between known Ctn elevations and negative histology. Such a request should be made promptly since the complete processing of the resected tissue is crucial for the diagnosis. It can certainly be assumed that some of the so-called “false-positive” cases of Ctn screenings or stimulation tests repeatedly described in the literature are due to undetected primary tumors—depending on the processing technique and expertise of the pathologist involved.

### 4.4. Limitations of Our Study

Since this is a retrospective study, the well-known limitations of retrospective data collection must be taken into account, such as the problem of selection bias or the lack of consideration of confounders. However, as far as the risk of sample bias is concerned, this can be classified as low because—as described in the methods section—a routine Ctn determination was actually initiated for all nodules that were at least 2 mm in size, without considering further nodule characteristics.

The data we obtained reflect the situation in a medical practice for nuclear medicine that closely works with relevant experts from other disciplines (practice for endocrinology, endocrine surgery, and pathology), i.e., as an interdisciplinary medical center. For most patients in the region, the practice is the primary point of contact for thyroid diagnostics by specialist doctors; patients are usually referred by their general practitioners. Thus, a certain preselection of patients cannot be ruled out; however, the patient population is significantly less preselected than at a university hospital.

As far as confounders for the results of the CTn screening are concerned, age, sex, family history, and genetic predisposition (here in particular mutations in the RET proto-oncogene) could be considered. Patients with a personal or family history of thyroid carcinoma or MEN were excluded from the study, as were those with known mutations of the RET proto-oncogene. The significant differences between the sex distribution of the total of 12984 screened patients (m:f = 1:3.98) and the 16 ultimately confirmed MTC cases (m:f = 1:1.67) indicate that sex is a possible confounder. Other possible confounders such as age were not systematically recorded in the 12,777 patients with normal calcitonin values.

Apart from the problems of the histopathological “gold standard” mentioned above and false-negative cases that can only be inaccurately determined, the number of 83 patients in our study lacking follow-up data despite basal Ctn values above the sex-specific reference ranges is unsatisfactory. This high proportion illustrates the usefulness of an automatic recall system in institutions that carry out systematic Ctn screening. While such systems are provided in most standard practice management software solutions, they must be activated separately. However, this “dropout” rate could not be significantly reduced by targeted letters to the referring doctors and/or patients. Fortunately, only patients in our study were affected whose Ctn values were still below the currently recommended sex-specific intervention threshold values of 30 (females) or 60 (males) pg/mL, so the risk of C-cell neoplasia in these patients was very low.

## 5. Conclusions

The extrapolated MTC prevalence of 0.14% from the data presented here is significantly lower than described in early international screening studies. From our data presented here, we conclude that—apart from rare exceptions—a stimulation test should be omitted when using a decision-making concept based on sex-specific Ctn cut-off values. Ctn screening for MTC in outpatient practices with a thyroidal focus is effectively feasible if high quality standards exist (e.g., optimal pre-analytics and laboratory performance, expertise in the interpretation of increased Ctn values and the further procedures depending on them) and close interdisciplinary networks (nuclear medicine, endocrinology, specialized surgery, and pathology). If possible, an automatic recall system should be in place to ensure that further recommended measures are actually performed in the event of increased Ctn values. To increase the chance of detecting an MTC at an early stage to ensure a biochemical cure, we recommend performing the Ctn screening even in the presence of very small thyroid nodules and regardless of the sonographic appearance. The recommendation by the Thyroid Section of the DGE to make the subsequent procedures for Ctn screening mostly dependent on the level and course of the bCtn values (surgery should be performed when a “monitoring range” of 20–30 pg/mL in women and 30–60 pg/mL in men is exceeded) appears to be a pragmatic compromise between over-treatment and timely tumor detection. In our study, the stimulation tests did not prove to be superior to this concept.

## Figures and Tables

**Table 1:** Characteristics of all 20 patients within the screening period November 2008–November 2014 in whom, based on Ctn measurement, the indication for thyroidectomy was made due to the suspicion of C-cell neoplasia. bCtn = basal calcitonin (at the time of screening, Ctn elevations had been confirmed per a confirmation test with ICMA); sCt = stimulated calcitonin; Ca test = calcium gluconate test, Pg test = pentagastrin test; CEA↑ = carcinoembryonic antigen elevated; MTC = medullary thyroid carcinoma (with the patient ID number in our study); CCH = C-Cell hyperplasia; PTC = papillary thyroid carcinoma.

Sex m = Male f = Female	Age at Time of Surgery [Years]	bCtn IRMA [pg/mL]	Biochemical Reasons for the Indication for Thyroidectomy Because of a Strong Suspicion of C-cell Neoplasia	Histology
f	64	10.1	bCtn increasing to 25.1 pg/mL in 3.75 years, sCtn (Ca-test) peak >2000 pg/mL	MTC-16
f	29	14.6	bCtn increasing to 71.6 pg/mL in 5.1 years, sCtn (Ca-test) peak >2000 pg/mL, CEA↑	MTC-09
m	65	17.8	bCtn increasing to 31.7 pg/mL in 8 months, sCtn (Ca-test) peak 703 pg/mL	PTC
f	45	19.3	bCtn moderately increased, sCtn (Ca-test) peak 612 pg/mL	MTC-03
m	73	30.5	bCtn moderately increased, sCtn (Ca-test) peak 1616 pg/mL, CEA↑	MTC-04
f	47	31.9	bCtn moderately increased, sCtn (Pg-test) peak 1189 pg/mL	MTC-11
m	57	35.5	bCtn moderately increased, sCtn (Ca-test) peak >2000 pg/mL	CCH
m	69	47.2	bCtn moderately increased, sCtn (Pg-test) peak 450 pg/mL, CEA↑	CCH
m	77	49.4	bCtn moderately increased, sCtn (Ca-test) peak 1517 pg/mL, CEA↑	MTC-13
f	45	51.6	bCtn markedly increased	CCH
f	38	53.0	bCtn markedly increased, sCtn (Ca-test) peak >2000 pg/mL	MTC-01
m	44	90.4	bCtn markedly increased, sCtn (Ca-test) peak 1336 pg/mL	MTC-06
f	78	128	bCtn extremely increased, sCtn (Ca-test) peak >2000 pg/mL	MTC-12
m	47	227	bCtn extremely increased, sCtn (Ca-test) peak 459 pg/mL	MTC-10
f	67	280	bCtn extremely increased, sCtn (Pg-test) peak 5347 pg/mL, CEA↑	MTC-08
m	72	291	bCtn extremely increased, sCtn (Ca-test) peak >2000 pg/mL, CEA↑	MTC-14
f	39	838	bCtn extremely increased, CEA↑	MTC-07
f	70	868	bCtn extremely increased, sCtn (Ca-test) peak >2000 pg/mL, CEA↑	MTC-02
m	72	1132	bCtn extremely increased, CEA↑	MTC-05
f	76	1276	bCtn extremely increased, CEA↑	MTC-15

## Data Availability

Data are available from the authors upon reasonable request. This paper contains substantial data from the doctoral thesis of Mirka Fries.

## Supplementary Materials

The following supporting information can be downloaded at https://www.mdpi.com/article/10.3390/cancers15082333/s1. Table S1: Characteristics of all 16 patients with histologically confirmed MTC that was detected by Ctn screening; Table S2: Overview of large international studies on routine Ctn measurement in nodular thyroid disease (only studies with at least 1000 patients). References [61,62,63] are cited in Supplementary Materials.
