# Supplementary material for: Update on Calcitonin Screening for Medullary Thyroid Carcinoma and the Results of a Retrospective Analysis of 12,984 Patients with Thyroid Nodules
Source: Cancers (Basel). 2023 Apr 17;15(8):2333. doi: 10.3390/cancers15082333 (PMC10136489; doi:10.3390/cancers15082333)
Supplement: Supplementary file 1 [file cancers-15-02333-s001.zip › Table S1.pdf]

**Suppl. Table S1.** Characteristics of all 16 patients with histologically confirmed MTC that was detected by Ctn screening. *external laboratory; stimulation test: Ca=calcium glucoronate, Pg=pentagastrin; n.d.= not determined

| ID | sex (f/m)<br>age at surgery [y] | ultrasonographical appearance of the MTC | Ctn (IRMA) at primary screening [pg/ml] | Ctn (ICMA) at primary screening [pg/ml] | CEA [ng/ml] | stimulation test: Ca / Pg (CTn peak [pg/ml]) | pre-op. Ctn [pg/ml] (time since primary screening) |
|---|---|---|---|---|---|---|---|
| MTC-01 | f<br>38 | MTC could not be clearly assigned to a nodule | 53.0 | 65.2 | 1.3 | Ca<br>(>2000) | 45.2<br>(8 months) |
| MTC-02 | f<br>70 | bilateral hypoechoic nodules with well-defined margins up to 1.2cm, part. microcalcifications | 868 | 821 | 16.6 | Ca<br>(>2000) | 1156<br>(2 months) |
| MTC-03 | f<br>45 | MTC could not be clearly assigned to a nodule | 19.3 | 31.6 | 3.1 | Ca<br>(612) | 29.9<br>(1.5 months) |
| MTC-04 | m<br>73 | MTC could not be clearly assigned to a nodule | 30.5 | 40.0 | 6.2 | Ca<br>(1616) | 54.0<br>(1 month) |
| MTC-05 | m<br>72 | 2.4x1.8x3.6cm isoechoic nodule with well-defined margins, small cystic internal structures, no calcifications | 1132 | 1022 | 22.2 | n.d. | 1022<br>(0 months) |
| MTC-06 | m<br>44 | MTC could not be clearly assigned to a nodule | 90.4 | 83.3 | 0.7 | Ca<br>(1363) | 120<br>(1,5 months) |
| MTC-07 | f<br>39 | 1.1x1.5x2.8cm hypoechoic nodule with well-defined margins | 838 | 632 | 36.7 | n.d. | 632<br>(0 months) |
| MTC-08 | f<br>67 | 0.7x1.0x1.0cm hypoechoic nodule with well-defined margins | 280 | 268 | 5.4 | Pg<br>(5347) | 268<br>(1 month) |
| MTC-09 | f<br>29 | 0.7x0.5x0.9cm hypoechoic nodule with well-defined margins, without calcifications | 14.6 | n.d. | 4.2 | Ca<br>(>2000) | 71.6<br>(5 years, 1 month) |
| MTC-10 | m<br>47 | 1.1x0.9x1.1cm hypoechoic nodule with well-defined margins | 227 | 81.1 | 1.5 | Ca<br>(459) | 108<br>(1 month) |
| MTC-11 | f<br>47 | MTC could not be clearly assigned to a nodule | 31.9 | n.d. | n.d. | Pg<br>(1189) | 28.0<br>(2 months) |
| MTC-12 | f<br>78 | 0.9x0.5x0.9cm hypoechoic nodule with well-defined margins and microcalcifications | 128 | 92 | 3 | Ca<br>(>2000) | 126<br>(1.5 months) |
| MTC-13 | m<br>77 | MTC could not be clearly assigned to a nodule | 49.4 | 41.2 | 4.1 | Ca<br>(1517) | 48.4<br>(1 month) |
| MTC-14 | m<br>72 | 1.4x1.4x2.3cm hypoechoic nodule with well-defined margins, small cystic structures, micro- and macrocalcifiations | 291 | 266 | 7.9 | Ca<br>(>2000) | 345<br>(1 month) |
| MTC-15 | f<br>76 | 2.1x2.6x1.3cm hypoechoic nodule with macrocalcfications and cystic structures | 1276 | 1312 | 42.2 | n.d. | 1312<br>(0 months) |
| MTC-16 | f<br>64 | MTC could not be clearly assigned to a nodule | 10.1 | n.d. | n.d. | Ca<br>(>2000) | 25.1<br>(3 years 9 months) |
