# Supplementary material for: Update on Calcitonin Screening for Medullary Thyroid Carcinoma and the Results of a Retrospective Analysis of 12,984 Patients with Thyroid Nodules
Source: Cancers (Basel). 2023 Apr 17;15(8):2333. doi: 10.3390/cancers15082333 (PMC10136489; doi:10.3390/cancers15082333)
Supplement: Supplementary file 1 [file cancers-15-02333-s001.zip › Table S2.pdf]

Suppl. Table S2. Overview of large international studies on routine Ctn measurement in nodular thyroid disease (only studies with at least 1000 patients).
Abbreviations: AFTN=autonomous functioning thyroid nodule; AIT=autoimmune thyroiditis; f=females; GD=Grave's disease; ICMA=immunochemiluminometric assay; IRMA=immunoradiometric assay; m=males; PPI=proton pump inhibitors. *1 additional patient had been diagnosed with MEN but refused thyroidectomy (Ctn 81.9 pg/ml); **including 2 histologically discovered MTC cases without elevated basal Ctn values.

first author publication year	country (region)	recruitment period	follow-up period	patients total	m:f	age [yrs.]	inclusion criteria	exclusion criteria	Assay	Basal Ctn cutoff [pg/ml]	Basal Ctn patients >cutoff n (%)	patients with elevated Ctn lost to follow-up or with incomplete data	MTC prevalence	proportion of sporadic MTC
Hahm 2001 [34]	Korea (Seoul)	6/1998-1999	not described	1,448	1:4.08	14-86	nodular thyroid disease	not specified	IRMA (Medgenix CT-U.S., BioSource)	10 (m/f)	56 (3.9 %)	not described	10/1,448 (0.69%)	80 %
Hatzl-Griesenhofer 2002 [61]	Austria (Linz)	1/1999-12/2001	not described	3,899	1:3.72	6-90	nodular thyroid disease	not specified	ICMA (Nichols Institute Diagnostic)	4.6 (f) 11.5 (m)	230 (5.9%) f:140 (4.6%) m:90 (10.9%)	23	12/3,899 (0.31%)	75 %
Elisei 2004 [4]	Italy (Sienna)	1991-1998	3 - 6 yrs.	10,864	1:4.00	12-82	nodular thyroid disease	not specified	IRMA (ELSA hCT, CIS-Biointernational)	20 (m/f)	47 (0.4%)	1	40/10,864 (0.37%)	80 %
Vierhapper 2005 [35]	Austria (Vienna)	8/1994-8/2004	not described	10,157	1:3.97	not reported	nodular thyroid disease	known Ctn elevation or C-cell neoplasia	1994-1999 IRMA (CIS-BioInt) 1994-2004 ICMA (Nichols Institute Diagnostics)	10 (m/f)	507 (5.0%)	18	32/10,158 (0.32%)	91 %
Papi 2006 [62]	Italy (Rome/ Modena)	4/2003-3/2004	not described	1425	1:4.07	18-91	nodular thyroid disease (non-palpable nodules <10 mm only in case of suspicious ultrasonogr. features)	known Ctn elevation or C-cell neoplasia, family history of MEN, patients in follow-up for thyroid diseases	ICMA (Nichols Institute Diagnostics)	5 (m/f)	23 (1.6%)	not described	9/1,425 (0.63%)	100 %
Costante 2007 [36]	Italy (various regions)	1/2001-12/2004	2 - 4 years	5,817	1:4.24	11-72	nodular thyroid disease	known MTC or family history of MTC, renal failure	ICMA (Nichols Advantage, Nichols Institute Diagnostics)	10 (m/f)	282 (4.8%)	45	15/5,817 (0.26%)	not reported
Rink 2009 [27]	Germany (Rhine-Main)	7/2000-10/2006	up to 87 months	21,928	1:3.32	8-97	nodular thyroid disease	not specified	IRMA (1. Calcitonin-IRMA, IBL; 2. Calcitonin-IRMA magnum, Medipan)	10 (m/f)	885 (4.0%)	509	28/21,928 (0.13%)	80 %

first author publication year	country (region)	recruitment period	follow-up period	patients total	m:f	age [yrs.]	inclusion criteria	exclusion criteria	Assay	Basal Ctn cutoff [pg/ml]	Basal Ctn patients >cutoff n (%)	patients with elevated Ctn lost to follow-up or with incomplete data	MTC prevalence	proportion of sporadic MTC
Herrmann 2010 [28]	Germany (Bochum)	6/2005-9/2009	not described	1,007	1:1.29	55±14	nodular thyroid disease	known Ctn elevation, impaired renal function, GD, AIT, pseudo-hypoparathyroidism, alcohol or drug abuse, PPI	ICMA (Immulite 2000, Siemens)	10 (m/f)	17 (1.7%)	none	2/1,007 (0.2%)	100 %
Grani 2012 [37]	Italy (Rome)	5/2005-2/2010	up to 36 months	1,073	1:4.96	56±13	patientes referred to FNAC because of suspicious nodules	not specified	ICMA (automated two-site immunoassay)	10 (m/f)	41 (3.8%)	2	2*/1,073 (0.19%)	not reported
Schneider 2012 [29]	Germany (Cologne)	1/2004-12/2010	up to 1 year	11,270	not reported	not reported	nodules ≥2 mm	MTC or family story of MTC, renal insufficiency, bacterial infection, alcohol abuse, PPI therapy, GD or AIT	ICMA (Immulite 2000, Siemens)	13 (m/f)	32 (0.3%)	2	12**/11,270 (0.11%)	not reported
Giovanella 2013 [38]	Switzerland (Bellinzona)	1/2008-12/2010	24 – 35 months	1,236	1:1.20	53±17	nodular thyroid disease	pulmonary or pancreatic tumours, kidney failure, sepsis, alcohol abuse, smoking, PPI therapy, AFTN, GD or AIT	ICMA (Immulite 2000, Siemens)	10 (m/f)	14 (1.1%)	none	2/1,236 (0.16%)	100 %
Silvestre 2019 [39]	Portugal (Lisboa region)	1/2011-12/2015	not described	1504	not reported	not reported	nodular thyroid disease	not specified	ICMA (Immulite 2000, Siemens)	10 (m/f)	69 (4.6%)	49	12/1,504 (0.80%)	86 %
Storani 2019 [63]	Argentinia (Buenos Aires)	1/2009-6/2017	up to 6 yrs.	1,017	1:7.07	18-88	nodules >2 mm	known C-cell neoplasia, chronic kidney failure, PPI therapy	ICMA (Immulite 2000, Siemens)	12 (f) 18 (m)	4 (0.4%)	none	2/1,017 (0.2%)	50 %
present study	Germany (Rhine-Ruhr)	11/2009-11/2014	up to 13 yrs.	12,984	1:3.98	18-90	nodules >2 mm	known Ctn elevation or C-cell neoplasia, advanced kidney disease	IRMA (SELco Calcitonin, Medipan)	10 (f) 15 (m)	207 (1.6%)	83	16/12,984 (0.12%)	82 %
